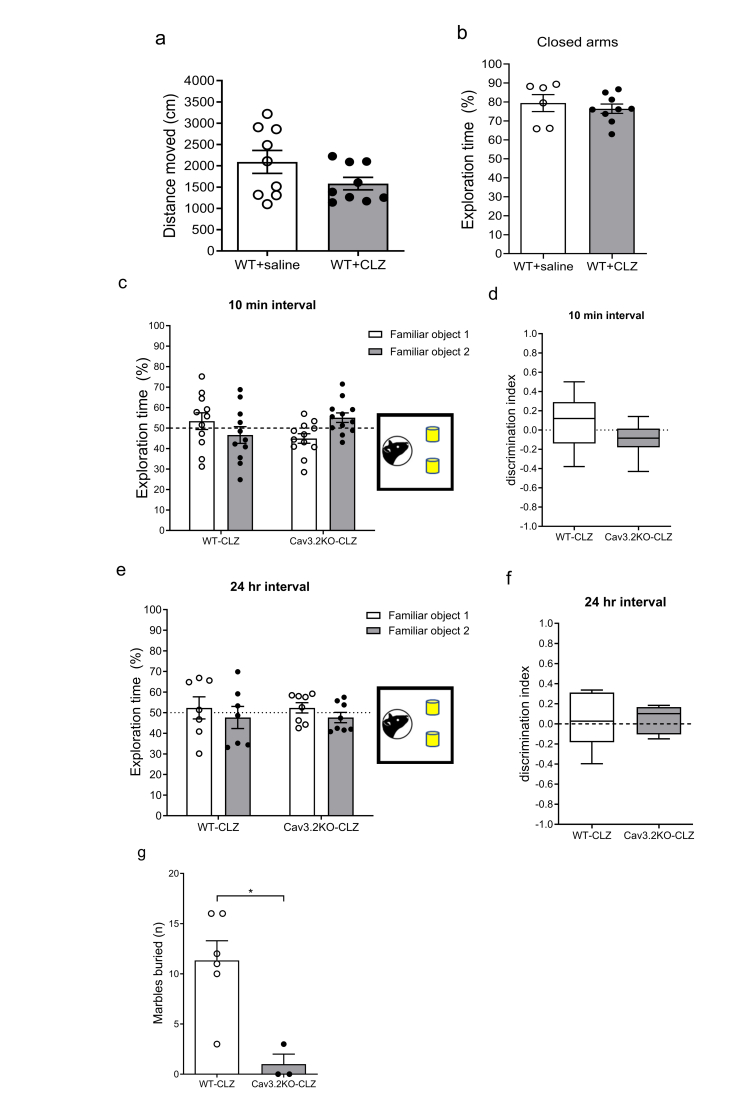# Activation of GABA transmission by clonazepam reverses the autistic-like phenotypes of the Cav3.2 knockout mice

**DOI:** 10.1016/j.neurot.2025.e00761

**Published:** 2025-10-08

**Authors:** Shao-Feng Liang, Yang Ming, Hsien-Ting Huang, Raymond Y. Lo, Supin Chompoopong, Chien-Chang Chen, Ingrid Y. Liu

**Affiliations:** a Doctoral Degree Program in Translational Medicine, Tzu Chi University and Academia Sinica, Taiwan; b Institute of Biomedical Sciences, Academia Sinica, Taipei, Taiwan; c Department of Molecular Biology and Human Genetics, Tzu Chi University, Hualien, Taiwan; d Okinawa Institute of Science and Technology Graduate University, Okinawa, Japan; e Institute of Medical Sciences, Tzu Chi University, Hualien, Taiwan; f Department of Neurology, Tzu Chi Hospital, Hualien, Taiwan; g Department of Anatomy, Faculty of Medicine Siriraj Hospital, Mahidol University, Bangkok, Thailand

**Keywords:** Autism spectrum disorder, *CACNA1H*, Clonazepam, Social behavior, GABA

## Abstract

Autism spectrum disorder (ASD) is a neurodevelopmental disorder characterized by social and communication deficits, accompanied by restricted and repetitive behaviors. ASD is a lifelong condition that causes a heavy medical and societal burden. To date, there are no disease-modifying, mechanism-targeted treatments approved for core ASD symptoms. In human studies, loss-of-function mutations in the *CACNA1H* gene, which encodes the T type Cav3.2 calcium channel, have been associated with ASD. However, animal and molecular studies investigating the underlying mechanism in ASD patients with *CACNA1H* mutations are lacking. In this study, we performed a series of behavioral assays to phenotype the Cav3.2 systemic knockout (Cav3.2KO) mice. The Cav3.2KO mice exhibited ASD-like behaviors, including impaired social novelty, increased self-grooming behavior, and deficits in recognition and retrieval of fear memory. Notably, enhancing γ-aminobutyric acid (GABA) signaling via administration a low-dose of clonazepam (CLZ) rescued these behavioral impairments in the Cav3.2KO mice. Furthermore, we found that the intrinsic GABA level was significantly reduced in the frontal cortex of Cav3.2KO mice, suggesting that GABA transmission was impaired in the Cav3.2KO mice. Together, our findings suggest that loss-of-function in the Cav3.2 channel contributes to ASD-like phenotypes through disrupted GABAergic signaling and that pharmacological enhancement of GABAergic signaling may offer a potential therapeutic approach for individuals with ASD carrying the *CACNA1H* mutations.

## Introduction

Autism spectrum disorder (ASD) is a neuropsychiatric disorder characterized by developmental abnormalities, including impaired social communication, language deficits, and restricted, repetitive patterns of behaviors or interests [1,2]. The term “spectrum” reflects the wide variability in symptoms and severity, which can include intellectual disability, epilepsy, sleep disorder, gastrointestinal disturbances, anxiety, and motor impairment [[1], [2], [3], [4]]. Given this heterogeneity, personalized precision medicine approaches tailored to the specific needs of each individual with ASD are essential [5]. The etiology of ASD is multifactorial, involving both genetic and environmental factors. The heritability of ASD ranges from approximately 40 ​%–80 ​% [6]. To date, hundreds of genes have been linked to ASD. Despite these insights, the U.S. Food and Drug Administration has approved only two drugs for treating irritability associated with ASD: risperidone [7] and aripiprazole [8]. Therefore, the development of disease-modifying treatments targeting the underlying mechanisms of core ASD symptoms is imperative.

Previous studies have revealed that mutations in voltage-gated calcium channel (VGCC) genes are associated with various neurological disorders, including schizophrenia, bipolar disorder, epilepsy, and ASD [9,10]. Among the VGCC genes, the *CACNA1H* gene, which encoded the low-voltage-activated T-type Cav3.2 calcium channel, has been reported as a susceptibility gene for ASD. In 2006, Splawski et al. [11] identified missense mutations in the *CACNA1H* gene in 6 of 461 individuals with ASD. Functional analysis of these mutant channels revealed a reduction in the current peak and less voltage sensitivity, suggesting decreased channel activity that may disrupt neuronal function. Over the past decade, several studies have reported loss-of-function *CACNA1H* variants in individuals with ASD, found across different ethnic backgrounds and in both males and females [[12], [13], [14], [15], [16], [17]]. Notably, the *CACNA1H* gene is classified as a strong ASD candidate gene in the SFARI Gene database (SFARI score ​= ​2, https://gene.sfari.org/database/human-gene/CACNA1H). Despite growing evidence of *CACNA1H* variants in ASD, many studies have been limited to variant identification, with few addressing the mechanistic underpinnings or potential therapeutic approaches. Thus, our work aims to bridge this gap by investigating the functional consequences of Cav3.2 channel loss and exploring its relevance to ASD pathophysiology and potential therapeutic targets.

Previous studies have shown that individuals with ASD exhibit an altered balance between excitatory and inhibitory neurotransmission (E/I imbalance), characterized by elevated excitatory over inhibitory transmission [[18], [19], [20]]. This has led some scientists to hypothesize that increased excitation or decreased inhibition in sensory, memory, social, and emotional systems may underlie the pathogenesis of ASD [21,22]. In addition, several well-established transgenic mouse models of ASD also display E/I imbalance [23,24]. Notably, pharmacological enhancement of GABAergic transmission with low-dose clonazepam (CLZ), a positive allosteric modulator of γ-aminobutyric acid type A receptors (GABA_A_Rs), has been shown to rescue impaired social behaviors and memory performance in ASD mouse models [[25], [26], [27], [28], [29]]. Together, boosting GABAergic signaling may represent a potential therapeutic strategy for alleviating core symptoms of ASD [30,31].

Given that loss-of-function *CACNA1H* variants are strongly associated with ASD, we employed Cav3.2 knockout (Cav3.2KO) mice in this study. Previous studies have demonstrated that Cav3.2KO mice exhibit a range of behavioral abnormalities, such as impaired recognition memory [32], deficits in context-cued trace fear conditioning [33,34], and aberrant social behavior [35,36], all of which reflect core and associated features of ASD. Additionally, Arshaad et al. [37] demonstrated that Cav3.2KO mice exhibit reduced transcriptional levels of GABA_A_ receptor δ subunit (*Gabrd*) and the GABA_B_ receptor B1 subunit (*Gabbr1*) in hippocampal tissue. Moreover, we found a reduction in GABA_A_ receptor β2 subunit (*Gabrb2*) transcripts in the right hippocampus after contextual trace fear conditioning [38]. Furthermore, Cav3.2 channels are expressed in GABAergic neurons [39,40] and play a role in regulating neuronal excitability [41,42]. Based on these findings, we hypothesized that loss of Cav3.2 channels may disrupt GABAergic signaling, resulting in behavioral deficits, and that these deficits can be rescued by enhancing GABAergic transmission.

Here, we conducted a series of behavioral tests in the Cav3.2KO mice and *Cacna1h*^+/+^ wild type (WT) mice. We observed that Cav3.2KO mice displayed normal locomotor activity, olfactory function, and anxiety levels, but showed behavioral deficits in self-grooming, social novelty, novel object recognition, and contextual fear memory retrieval. These findings suggest that Cav3.2KO mice exhibit ASD-like behaviors. To determine whether enhancing GABAergic signaling could mitigate these deficits, we next administered a low-dose CLZ to both Cav3.2KO and WT mice. The CLZ treatment rescued the behavioral abnormalities observed in Cav3.2KO mice without inducing sedative effects. Furthermore, we found that the GABA level was reduced in the frontal cortex (FC) of Cav3.2KO mice as determined by enzyme-linked immunosorbent assay (ELISA). Based on these observations, we suggest that enhancement of GABA signaling could be a promising therapeutic approach for individuals with ASD carrying the *CACNA1H* mutation.

## Materials and Methods

### Mice

The homozygous Cav3.2 knockout (Cav3.2KO) mice on a C57BL/6J background (RRID: IMSR_JAX:013770; MGI:4836206) were provided and originated from Dr. Chien-Chang Chen as previously described [43]. In these mice, exon 6 of *cacna1h*, which encodes residues 216 through 267, was targeted for deletion and replaced with a floxed neo cassette inserted by homologous recombination. The Cav3.2KO mice were bred from *Cacna1h*^*+/−*^ ​× ​*Cacna1h*^*+/−*^ and *Cacna1h*^+/−^ ​× ​*Cacna1h*^−/−^ parents. The mice used for all the behavioral and molecular analyses were 8- to 12-week-old males. After postnatal day (P) 7–14, each mouse was genotyped by polymerase chain reaction (PCR) amplification of exon 6 of *cacna1h*. The primers used for PCR amplification of exon 6 were as follows: primer#1–5′-ATTCAAGGGCTTCCACAGGGTA-3′, primer#2–5′-CATCTCAGGGCCTCTGGACCAC-3′, and primer#3–5′-GCTAAAGCGCATGCTCCAGACTG-3'. The size of the WT and mutant amplicons are 480 and 330 base pairs (Supplementary Fig. 1). The mice were weaned at P21 and housed by sex and genotype in specific pathogen-free conditions with food and water *ad libitum* under a 12-h light/dark cycle (7:00∼19:00 light period). Each cage housed 2–5 animals. Littermate WT controls were used in three-chamber social test, self-grooming, elevated plus maze, marble burying test, and contextual test of trace fear conditioning. C57BL/6J WT controls purchased from the National Laboratory Animal Center in Taiwan (MGI:5699857) were used in open field test, nest building test, buried food test, reciprocal social interaction test, five-trial social memory test, and GABA measurement. All procedures were approved by the Institutional Animal Care and Use Committee of Tzu Chi University (Project No. 108029). Animal care and handling were performed according to the guidelines of the Institutional Animal Care and Use Committee of Tzu Chi University.

### Drug Administration

Clonazepam (Rivotril ® 0.5 ​mg/T, Roche) tablets were ground and suspended in 0.9 ​% physiological saline to a final concentration of 0.05 ​mg/mL. Mice received an intraperitoneal (i.p.) injection of clonazepam (0.05 ​mg/kg), or the same volume of saline, 30 ​min before all the behavioral tests.

### Mouse behaviors

Behavioral experiments were conducted in an isolated, sound-attenuated, temperature- and humidity-controlled room under consistent lighting conditions between 09:00 and 18:00. All behavioral apparatuses were cleaned with 75 ​% ethanol between subjects to eliminate olfactory cues. Mice were habituated to the testing room for at least 30 ​min prior to the onset of each behavioral test. A minimum inter-test interval of 24 ​h was maintained between assays to reduce carryover effects. The illumination level of the behavioral room was maintained at 75 lx.

#### Open field test

A mouse was placed on the wall-side of white plexiglass chamber (40 ​cm length ​× ​40 ​cm width ​× ​40 ​cm depth) and freely explored the arena for 10 ​min. Mouse movement was recorded by a webcam. The total distance moved, average velocity, and total time spent in the center (one ninth of total area) were analyzed using ANY-maze 6.0 software (Stoelting Co., USA).

#### Nest building test

Two grams of shredded printer paper strips (7 ​cm length ​× ​0.5 ​cm width) were dispersed in a new plexiglass home cage (30 ​cm length ​× ​15.5 ​cm width ​× ​13 ​cm depth). As previously described [44], at 18:00, each mouse was individually housed overnight with food and water *ad libitum* provided in the cage. After 14 ​h, a camera photographed the nest from the top of each cage. The open arena was conceptually divided into 6 regions for scoring. Points were assigned based on the degree of coverage within each region: 1 point for an uncovered region, 0.5 points if a region was covered by less than half of its area, and 0 points if a region was covered by more than half of its area. The nest score started with a baseline of 1 point for every mouse, with additional points accumulated based on the aforementioned criteria, yielding higher score for better nesting performance.

#### Buried food test

Each mouse was individually housed without food. After 20 ​h of food deprivation, the mouse was placed in the center of a plexiglass testing cage (30 ​cm length ​× ​15.5 ​cm width ​× ​13 ​cm depth) containing 3 ​cm of fresh bedding and allowed to explore freely for 5 ​min. A standard food chow was buried beneath 1 ​cm of bedding at a random site (left, middle, or right) near the wall of the test cage. Furthermore, 0.5 ​cm of fresh bedding was added to the test cage. Each mouse was then reintroduced into the center of the test cage with buried food chow. A webcam recorded the video from the side of the test cage. The time animals spent digging out the food chow was measured in seconds (latency), up to a maximum of 10 ​min.

#### Three-chamber social test

The three-chamber apparatus used in this study was a 50 ​cm length ​× ​25 ​cm width ​× ​25 ​cm depth plexiglass arena divided into three equally sized, interconnected chambers. The three-chamber assay consisted of three sessions: (1) *Habituation:* Subject mouse was placed in the center of the three-chambered apparatus, with two empty cylindrical wire containers positioned in the corner of both side chambers. They were allowed to freely explore for 10 ​min. (2) *Social interaction:* Subject mouse was returned to its home cage after habituation session. An age- and gender-matched WT male mouse (stranger 1) that had no prior contact with the subject mouse was placed in one of the two-wire containers. The subject mouse was then placed in the center and allowed to freely explore all three chambers for 10 ​min. (3) *Social novelty:* Subject mouse was returned to its home cage after social interaction session. Another WT male mouse (stranger 2) that had no prior contact with the subject mouse was placed in the wire container that had remained empty during the previous session. The subject mouse was then placed in the center and allowed to freely explore all three chambers for 10 ​min. The interval time between each session was 10 ​min. Mouse movement was recorded by a webcam, and the time spent in each chamber and the total distance moved were measured by video tracking system (Track Mot, Drinstrument, Taiwan). The percentage of exploration time was calculated using the formula: Exploration time % = (Time in specific chamber/Total exploration time) ​× ​100.

#### Self-grooming

Each mouse was individually placed into a novel plexiglass home cage (30 ​cm length ​× ​15.5 ​cm width ​× ​13 ​cm depth), approximately the same size as a standard home cage, for 10 ​min of habituation. During the subsequent 10 ​min, the total time spent grooming the face, head, body, and licking paws and legs was recorded and analyzed for each mouse.

#### Reciprocal social interaction test

Each mouse was individually housed before 4 ​h of testing. Subsequently, each mouse was introduced to an age- and gender-matched socially naïve mouse for 10 ​min of unrestricted interaction in a new plexiglass testing cage (30 ​cm length ​× ​15.5 ​cm width ​× ​13 ​cm depth). The behavior of each mouse was videotaped by a webcam. The frequency of social behaviors, including general sniffing, anogenital sniffing, following, and push-crawl behaviors, as well as non-social behaviors, including digging, rearing and self-grooming, were recorded.

#### Five-trial social recognition test

Each mouse was housed individually for 4 ​h prior to testing. The subject mouse was then placed in a white plexiglass chamber (40 ​cm length ​× ​40 ​cm width ​× ​40 ​cm depth) containing a cylindrical wired cup and allowed to freely explore for 5 ​min. A gender-matched juvenile mouse (4 weeks-old, referred to the familiar mouse) was placed inside the cylindrical wired cup. Four consecutive 5-min interaction trials, each separated by a 5 ​min inter-trial interval, were conducted for each subject mouse. In the 5th trial, the familiar mouse was replaced by a novel, gender-matched juvenile mouse. Sniff time was recorded by a stopwatch when the subject mouse oriented its nose within 1 ​cm of the cup containing the unfamiliar (novel) mouse.

#### Marble burying test

Mice were placed in the corner of a clear plexiglass cage (30 ​cm length ​× ​18.8 ​cm width ​× ​13.5 ​cm depth) containing 5 ​cm depth of fresh bedding with 5 rows of 4 glass marbles (1.5 ​cm diameter) evenly distributed on the surface. They were allowed to explore for 30 ​min without food and water. The number of marbles buried at least two-thirds of their surface covered by bedding was counted.

#### Elevated plus-maze

The elevated plus maze used in this study was elevated 60 ​cm from the floor and consisted of two opposite closed arms (25 ​cm length ​× ​5 ​cm width) surrounded by 15 ​cm high non-transparent walls, two opposing open arms (25 ​cm length ​× ​5 ​cm width), and a central square of 5 ​cm sides. The mouse was placed in the center of the maze, facing one of the open arms and allowed to explore the maze freely for 10 ​min. A video tracking system (Track Mot, Drinstrument, Taiwan) measured the times spent in the center, closed and open arms, and the total distance moved. The percentage of exploration time was calculated using the formula: Exploration time % = (Time in specific zone/Total exploration time) ​× ​100.

#### Novel object recognition test

A mouse was placed facing to the wall side of a white plexiglass chamber (40 ​cm length ​× ​40 ​cm width ​× ​40 ​cm depth) and allowed freely to explore the open field arena. The objects used had approximately dimensions of 4 ​cm length ​× ​4 ​cm width ​× ​7 ​cm height. Each mouse was habituated for 10 ​min per day for 2 days. On day 3, two identical objects were placed in opposite corners, 10 ​cm from the sidewalls. The mouse was then introduced into the wall side of the arena and allowed to freely explore the arena, including the two novel objects, for 10 ​min. After a 10 ​min interval, one object was replaced with a different novel object, which was similar in size but different in shape, color, and material from the previous object. The same mouse was placed into the wall side of the arena and allowed to freely explore the arena for 5 ​min. To test recognition memory, the 10 ​min interval was increased to 24 ​h. Mouse movement was recorded from above by a webcam and further analyzed with ANY-maze 6.0 software (Stoelting Co., USA). Data were excluded from the analysis if the total time of object exploration was less than 5 ​s. The percentage of exploration time was calculated using the formula: Exploration time % = (Time in specific zone/Total exploration time) ​× ​100. The discrimination index was calculated using the formula: Discrimination index ​= ​[novel (sec) ​− ​familiar (sec)/novel (sec) ​+ ​familiar (sec)].

#### Contextual fear conditioning

The protocol of trace fear conditioning followed a previously published study [34]. Freezing behavior was recorded by a digital video camera and analyzed with Freeze Scan 1.0 software (Clever Sys, Inc., Reston, VA, USA). After 24 ​h following the trace fear conditioning test, each mouse was placed in the same chamber used for training, without presentation of the tone cue and foot shock, for 6 ​min to assess contextual memory retrieval, as indicated by the degree of freezing behavior.

### GABA measurement by enzyme-linked immunosorbent assay (ELISA)

After the mice were sacrificed by decapitation, the frontal cortical and hippocampal tissues were dissected, weighed, and immediately placed in liquid nitrogen before storage at −80 ​°C. The tissue samples were then homogenized in phosphate-buffered saline (pH 7.4). The supernatant was collected after centrifugation at 3000 ​rpm for 20 ​min at 4 ​°C and stored at −80 ​°C for future use. Prior to ELISA, the total protein concentration in the supernatant was determined using the *DC* (detergent compatible) protein assay (#5000112, BIO-RED). ELISA results were normalized to total protein content and expressed as pg/mL/mg of total protein. The GABA concentration was measured using a QuickDetect™ GABA mouse ELISA kit (#E445-100, BioVision) according to the manufacturer's instructions. Each sample was run in duplicate.

### RNA extraction

After the mouse was sacrificed by decapitation, the frontal cortex and hippocampus tissue were immediately dissected and homogenized with a microcentrifuge pestle (PP Micro Centrifuge Sample Pestle, Foremost Product Intl., CA, USA) in an eppendorf tube containing 50 ​mg of tissue per 500 ​μL of NucleoZOL (MACHEREY NAGEL GmbH & Co. KG, Postfach, Düren, Germany, 740404.200) until the sample was uniformly dissociated. The lysate was processed for RNA extraction according to the manufacturer's protocol.

### Quantitative real-time polymerase chain reaction (qRT-PCR)

Two μg of total RNA from each sample was converted into first-strand complementary DNA (cDNA) using Invitrogen™ Reverse Transcription Kit (Invitrogen™, CA, USA). Real-time PCR was performed by adding 1 ​μg of the cDNA template to 5 ​μL of Power SYBR® Green PCR Master Mix (Thermo Fisher Scientific, USA) and 1 ​μL of 5 ​μM specific primers, with and nuclease-free water added to a final volume of 10 ​μL. Primers sequences are presented in Table 1. Real-time PCR was performed in QuantStudio™ 5 Real-Time PCR System (Thermo Fisher Scientific, USA) for 40 cycles, consisting of denaturation at 95 ​°C for 15 ​s, annealing at 63 ​°C for 15 ​s, and extension at 72 ​°C for 30 ​s. The results of qRT-PCR were analyzed using the threshold cycle (Ct) during the exponential phase, and the 2^−ΔΔCt^ method [45] was used to calculate relative mRNA expression of the targeted gene by normalizing to the internal control gene, *Gapdh*.

**Table 1:** The primer sets for qRT-PCR.

Gene	Sequence 5′-3′	Size (bp)	Accession No.
*Gad1*	Forward: TGGAGCTGGCTGATTACCTC Reverse: CCATGGTTGTTCCTGACTCC	210	NM_008077
*Gad2*	Forward: CCGAGAAGGCTATGAAATGG Reverse: CTTGTCCCCTAAGGGTTGGT	199	NM_008078
*Gapdh*	Forward: GGTCATCCCAGAGCTGAACG Reverse: TACTTGGCAGGTTTCTCCAGG	108	NM_001289726

### Western blotting

The frontal cortex and hippocampus tissue were immediately dissected and homogenized with a microcentrifuge pestle in the lysis buffer containing 0.1 ​M NaCl, 0.05 ​M Tris, 0.1 ​% Triton X 100, 1 ​% phosphatase inhibitor (Phosphatase Inhibitor Cocktail Set II, Calbiochem ®, Germany) and 2 ​% protease inhibitor (Protease Inhibitor Cocktail Set I, Calbiochem ®, Germany). Samples were incubated on ice for 30 ​min. The lysate was then centrifuged at 13,000 ​g for 20 ​min at 4 ​°C. The protein-containing supernatant was collected. Total protein was quantified using Bio-Rad Protein Assay Dye Reagent Concentrate (BIO-RAD, USA). Forty μg of protein sample was separated on a 10 ​% polyacrylamide gel and transferred on PVDF membrane in transfer buffer by running at 25 ​V overnight at 4 ​°C. Membranes were blocked in 5 ​% bovine serum albumin (BSA) in Tris-Buffered Saline with Tween 20 (TBST, 20 ​mM Tris and 150 ​mM NaCl pH7.4, 0.1 ​% v/v Tween 20) for 1 ​h at room temperature. They were then incubated overnight at 4 ​°C with the following primary antibodies diluted in TBST with 5 ​% BSA at the indicated dilution: GAD1 (MAB5406, Merck, 1:5000), GAD2 (MAB351, Merck, 1:1000), and horseradish peroxidase (HRP)-conjugated GAPDH (HRP-60004, Proteintech, 1:5000). A secondary antibody conjugated to HRP was diluted 1:5000 in TBST with 3 ​% BSA and added to membranes for 1–2 ​h at room temperature. Membranes were visualized using Amersham ECL Select™ Western Blotting Detection Reagent (cytiva, #RPN2235) and imaged using LAS-4000 mini imaging system (Fujifilm Life Science, USA).

### Statistical analysis

Statistical analyses were performed using GraphPad Prism 10 0.2 0.2 software. Results are presented as the means ​± ​SEMs. Data distribution was assessed via the Shapiro-Wilk normality test. Statistical significance was evaluated via unpaired *t*-test, unpaired Mann-Whitney tests, or two-way-analysis of variance (ANOVA) followed by Bonferroni's post hoc test with multiple statistical comparisons between groups. Multiplicity across behavior panels was controlled by grouping conceptually related endpoints and adjusting *p*-values accordingly using Bonferroni correction, as appropriate. Differences were defined as statistically significant as follows: ∗*p* ​< ​0.05, ∗∗*p* ​< ​0.01, ∗∗∗*p* ​< ​0.001 and ∗∗∗∗*p* ​< ​0.0001.

## Results

### The Cav3.2KO mice displayed ASD-like behaviors

To assess whether the Cav3.2KO mice exhibit ASD-like behaviors, we performed the three-chamber social test (3CT) and self-grooming test in both Cav3.2KO mice and WT mice. In the 3CT, both Cav3.2KO and WT mice spent more time in the chamber containing the first stranger mouse compared to the empty cage chamber (Fig. 1a). However, when a second, novel stranger mouse was placed in the previously unoccupied chamber, only WT mice showed a significant preference for interacting with the new, unfamiliar mouse. In contrast, Cav3.2KO mice failed to show this preference, indicating impaired social novelty (Fig. 1b).

**Fig. 1:**
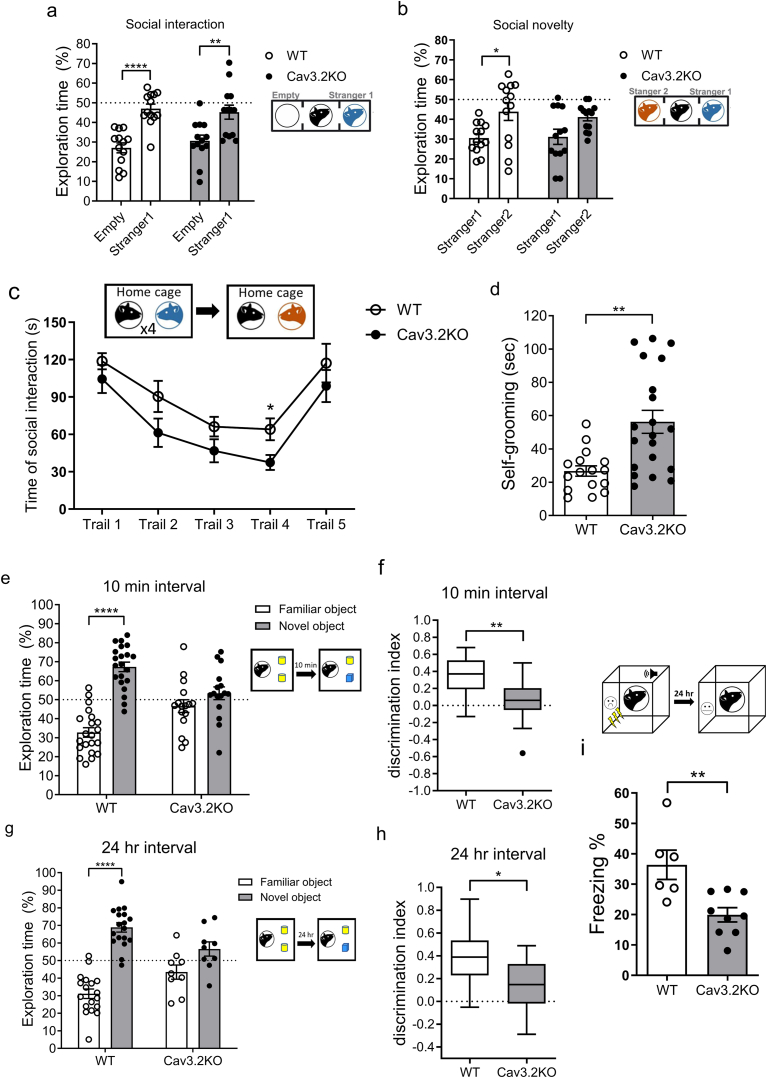
**Behavioral performance in the 3CT, self-grooming test, 5-trial social memory test, NORT, and contextual test of TFC in Cav3.2KO and WT mice** (a) Percentage of time spent exploring a novel mouse versus an empty wire container (*n* ​= ​13 for WT; *n* ​= ​13 for Cav3.2KO; two-way ANOVA with Bonferroni's multiple comparisons test, *F*_1,48 (interaction)_ ​= ​0.89, *p* ​= ​0.3495, η^2^ ​= ​0.010). (b) Percentage of time spent exploring a familiar versus a novel mouse in the 3CT (*n* ​= ​13 for WT; *n* ​= ​13 for Cav3.2KO; two-way ANOVA with Bonferroni's multiple comparisons test, *F*_1,48 (interaction)_ ​= ​0.26, *p* ​= ​0.6096, η^2^ ​= ​0.004). (c) Time spent in social interaction during the 5-trial social memory test (*n* ​= ​10 for WT; *n* ​= ​9 for Cav3.2KO; multiple unpaired *t*-test, *t*_(17)_ ​= ​2.49, *p* ​= ​0.0271, *Cohen's d* ​= ​1.125). (d) Total time spent on self-grooming (*n* ​= ​16 for WT; *n* ​= ​20 for Cav3.2KO; Mann-Whitney test, *p* ​= ​0.0015). (e) Percentage of time spent exploring a familiar object versus a novel object (two-way ANOVA with Bonferroni's multiple comparisons test, *F*_1,70 (interaction)_ ​= ​23.12, *p* ​< ​0.0001, η^2^ ​= ​0.160) and (f) discrimination index (unpaired *t*-test, *t*_(35)_ ​= ​3.39, *p* ​= ​0.0018, *Cohen's d* ​= ​1.111) in the 10 ​min interval protocol of the NORT (n ​= ​21 for WT; n ​= ​16 for Cav3.2KO). (g) Percentage of time spent exploring a familiar object versus novel object (two-way ANOVA with Bonferroni's multiple comparisons test, *F*_1,50 (interaction)_ ​= ​13.37, *p* ​= ​0.0006, η^2^ ​= ​0.111) and (h) discrimination index (unpaired *t*-test, *t*_(25)_ ​= ​2.59, *p* ​= ​0.0159, *Cohen's d* ​= ​1.044) in the 24 ​h interval protocol of the NORT (*n* ​= ​18 for WT; *n* ​= ​9 for Cav3.2KO). (i) Percentage of freezing behavior during the contextual test after 24 ​h TFC (*n* ​= ​6 for WT; *n* ​= ​9 for Cav3.2KO; unpaired *t*-test, *t*_(13)_ ​= ​3.41, *p* ​= ​0.0046, *Cohen's d* ​= ​1.698). ∗*p* ​< ​0.05, ∗∗*p* ​< ​0.01, and ∗∗∗∗*p* ​< ​0.0001.

To further investigate intrinsic social behavior, we performed a reciprocal social interaction test. The Cav3.2KO mice displayed a comparable number of social interactions to WT mice, including general sniffing, anogenital sniffing, push-crawl, and following behavior (Supplementary Fig. 2a). In the 5 trial-social recognition test, although the Cav3.2KO mice successfully recognized novel and gender-matched juvenile WT mice, they spent significantly less time interacting with familiar and gender-matched juvenile WT mice during the fourth trial (Fig. 1c). This suggests that Cav3.2KO mice display a reduced interest in familiar conspecifics despite repeated social encounters.

In addition, the Cav3.2KO mice displayed increased spontaneous self-grooming behavior compared to WT mice (Fig. 1d), a phenotype commonly interpreted as a repetitive behavior analogous to that seen in ASD [46]. Recent studies have reported that individuals with ASD often present not only with social impairments but also deficits in non-social memory, including working memory and episodic memory [47,48]. To address whether the Cav3.2KO mice exhibit impaired cognitive and memory functions, we performed a novel object recognition test (NORT) and a context-dependent fear-conditioning test. In the NORT, WT mice showed a clear preference for exploring the novel object, whereas Cav3.2KO mice did not discriminate between the familiar and novel objects (Fig. 1e, f, g, h and Supplementary Fig. 2b, c, d, e), suggesting impaired recognition memory. Additionally, the Cav3.2KO mice exhibited significantly reduced freezing behavior compared to WT mice when re-exposed to the conditioning chamber after 24 ​h of trace fear conditioning (Fig. 1i), indicating deficits in contextual fear memory retrieval. Taken together, these results suggest that the Cav3.2KO mice displayed ASD-like behaviors.

### The Cav3.2KO mice exhibited comparable locomotor activity, olfactory function, and nest-building ability to those of WT mice

Next, we assessed the locomotor activity of Cav3.2KO mice using the open field test (OFT). There were no significant differences in the total distance moved and average velocity between Cav3.2KO and WT mice (Fig. 2a, b, c), suggesting that loss of the Cav3.2 calcium channel does not affect locomotor function. Anxiety is one of the comorbidities of ASD [2,49]. To determine whether the Cav3.2KO mice exhibited anxiety-like behavior, we utilized both the OFT and the elevated plus-maze (EPM). In the OFT, the Cav3.2KO mice spent comparable time in the center zone relative to WT mice, indicating an anxiety-free phenotype (Fig. 2c and d). Similarly, in the EPM, Cav3.2KO mice spent a comparable amount of time in the closed arms as WT mice (Fig. 2e), further supporting that the absence of anxiety-related phenotypes.

**Fig. 2:**
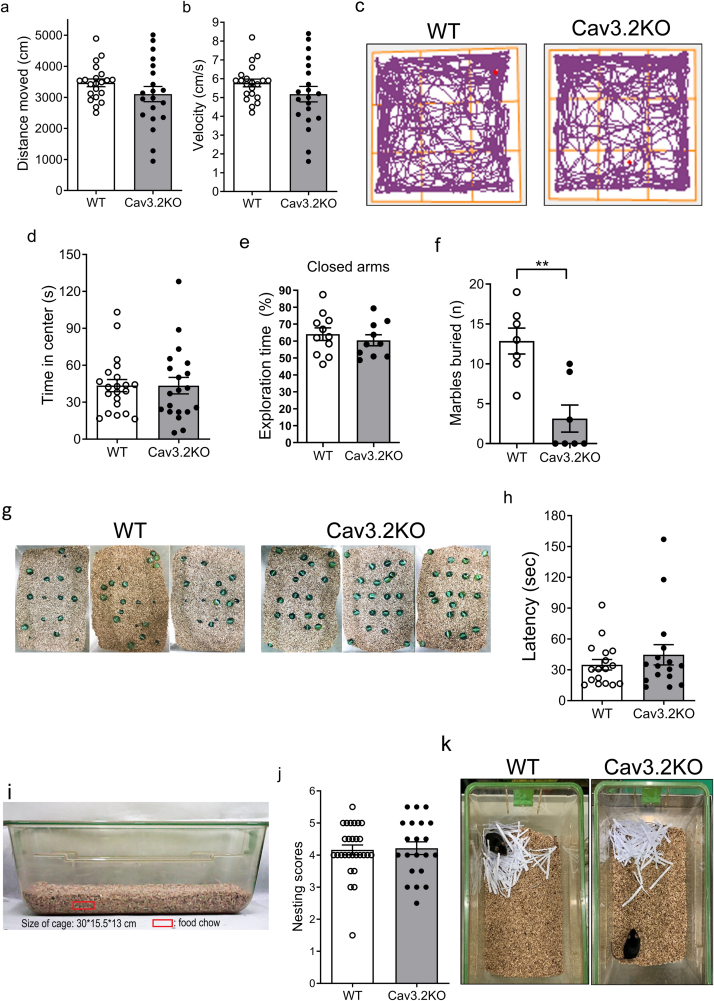
**Behavioral performance in the OFT, EPM, marble burying test, buried food test, and nest building test in Cav3.2KO and WT mice** (a) Distance moved (unpaired *t*-test, *t*_(39)_ ​= ​1.34, *p* ​= ​0.1880, *Cohen's d* ​= ​0.416) and (b) average velocity (unpaired *t*-test, *t*_(39)_ ​= ​1.31, *p* ​= ​0.1983, *Cohen's d* ​= ​0.406) in the OFT (*n* ​= ​20 for WT; *n* ​= ​21 for Cav3.2KO). (c) Representative locomotor tracings from the OFT. The red dot indicates the end point. (d) Time spent in the center of the open field apparatus (*n* ​= ​21 for WT; *n* ​= ​20 for Cav3.2KO; Mann-Whitney test, *p* ​= ​0.8872). (e) Percentage of time spent in the closed arms of the EPM (*n* ​= ​11 for WT; *n* ​= ​10 for Cav3.2KO; unpaired *t*-test, *t*_(19)_ ​= ​0.73, *p* ​= ​0.4726, *Cohen's d* ​= ​0.321). (f) Number of marbles buried (*n* ​= ​7 for WT; *n* ​= ​6 for Cav3.2KO; Mann-Whitney test, *p* ​= ​0.0029). (g) Representative images from the marble bury test after 20 ​min. (h) Latency of a food pellet to be uncovered (*n* ​= ​17 for WT; *n* ​= ​16 for Cav3.2KO; Mann-Whitney test, *p* ​= ​0.7550). (i) Diagram of the experimental setup for the buried food test. (j) Average nesting scores (*n* ​= ​21 for WT; *n* ​= ​28 for Cav3.2KO; Mann-Whitney test, *p* ​= ​0.8816). (k) Representative images of nests after 14 ​h ∗∗*p* ​< ​0.01.

Besides, we performed a marble burying test, which is commonly used to evaluate anxiety-like, compulsive, or repetitive behavior in rodents [50,51]. Intriguingly, the Cav3.2KO mice buried significantly fewer marbles compared to WT mice (Fig. 2f and g), suggesting that the Cav3.2KO mice had less defensive digging behavior.

Next, we performed the buried food test to examine olfactory function in both WT and Cav3.2KO mice. Mice were deprived of food pellets for 20 ​h to increase their motivation for food-seeking behavior. Both Cav3.2KO and WT mice presented similar percentages of body weight loss after food deprivation (Supplementary Fig. 3a and b). The results demonstrated that the Cav3.2KO mice spent comparable to WT mice in uncovering the buried food pellet (Fig. 2h and i), indicating intact olfactory sensitivity.

Finally, to assess innate behaviors related to home-cage activity, we performed a nest-building test. We scattered the stripped papers in the experimental cage 1 ​h before the onset of dark phase (Supplementary Fig. 3c), and nests were evaluated 14 ​h later. The Cav3.2KO mice constructed nests of comparable quality to those of WT mice (Fig. 2j and k), suggesting preserved species-typical behaviors.

### Low-dose clonazepam treatment rescued impaired behaviors in the Cav3.2KO mice

Previous studies have shown that enhancement of GABAergic transmission via low-dose CLZ can rescue behavioral deficits, including social interaction, social novelty and memory, in several animal models of ASD [25,26,28]. Additionally, transcriptomic analysis of Cav3.2KO mice have revealed reduced expression of certain GABA_A_Rs [37,38]. Based on these findings, we hypothesized that CLZ might alleviate ASD-like behaviors in Cav3.2KO mice.

To test this hypothesis, we administered a low-dose CLZ (0.05 ​mg/kg, i.p.) [25,26] into both Cav3.2KO and WT mice, followed by a series of behavioral assessments conducted 30 ​min post-injection. Notably, administration of CLZ at this dosage did not produce significant sedative or anxiolytic effects in WT mice compared to saline controls (Supplementary Fig. 4a and b).

We first performed 3CT and found that CLZ treatment did not affect social interaction in either WT or the Cav3.2KO mice (Fig. 3a). However, CLZ treatment successfully rescued the impaired social novelty recognition in Cav3.2KO mice, restoring their preference for the second, unfamiliar stranger mouse (Fig. 3b). Additionally, the elevated self-grooming behavior observed in Cav3.2KO mice was significantly reduced following CLZ treatment, reaching levels comparable to those of untreated WT mice (Fig. 1, Fig. 3d). Furthermore, CLZ treatment also normalized recognition and memory performance in Cav3.2KO mice, as demonstrated by novel object recognition (Fig. 3d, e, f, g and Supplementary Fig. 4c, d, e, f) and retrieval of contextual fear memory (Fig. 3h). However, CLZ treatment had no effect on the reduced marble-burying behavior in Cav3.2KO mice (Supplementary Fig. 4g).

**Fig. 3:**
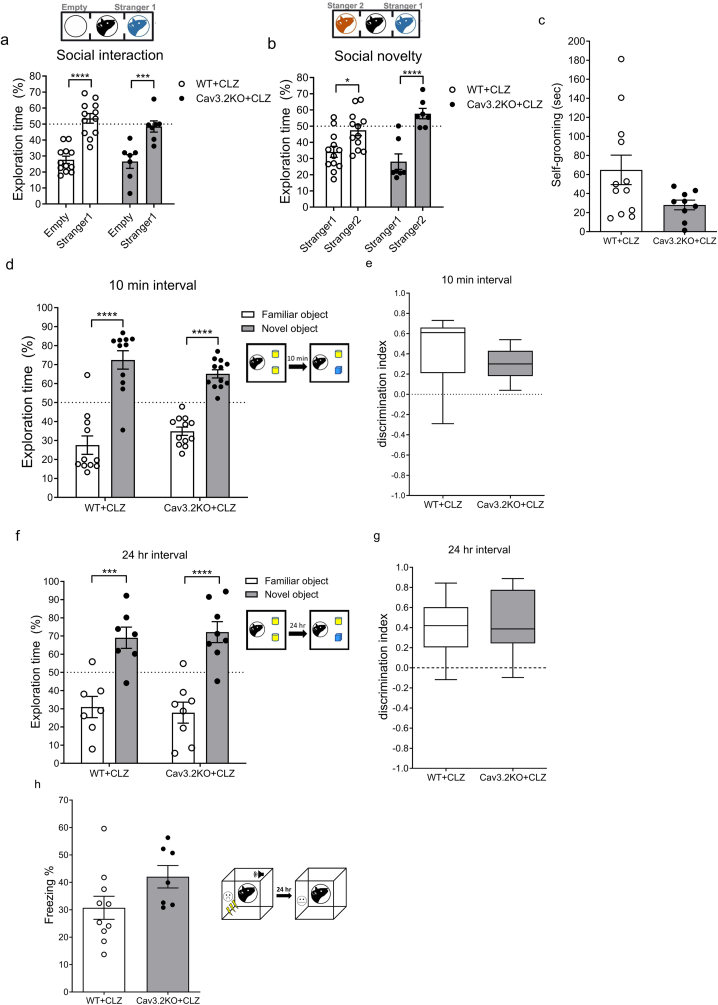
**Effect of Clonazepam treatment on 3CT, self-grooming test, NORT, and contextual test of TFC in Cav3.2KO and WT mice** (a) Percentage of time spent exploring a novel mouse versus an empty wire container in the 3CT after 30 ​min of clonazepam treatment (*n* ​= ​12 for WT; *n* ​= ​7 for Cav3.2KO; two-way ANOVA with Bonferroni's multiple comparisons test, *F*_1,34 (interaction)_ ​= ​0.38, *p* ​= ​0.5425, η^2^ ​= ​0.004). (b) Percentage of time spent exploring a familiar mouse versus a novel mouse in the 3CT after 30 ​min of clonazepam treatment (*n* ​= ​12 for WT; *n* ​= ​7 for Cav3.2KO; two-way ANOVA with Bonferroni's multiple comparisons test, *F*_1,34 (interaction)_ ​= ​4.58, *p* ​= ​0.0396, η^2^ ​= ​0.065). (c) Total time spent on self-grooming after 30 ​min of clonazepam treatment (*n* ​= ​12 for WT; *n* ​= ​9 for Cav3.2KO; Mann-Whitney test, *p* ​= ​0.0955). (d) Percentage of time spent exploring a familiar object versus a novel object (two-way ANOVA with Bonferroni's multiple comparisons test, *F*_1,42 (interaction)_ ​= ​4.04, *p* ​= ​0.0510, η^2^ ​= ​0.027) and (e) discrimination index (Mann-Whitney test, *p* ​= ​0.0818) in the 10 ​min interval protocol of the NORT after 30 ​min of clonazepam treatment (*n* ​= ​11 for WT; *n* ​= ​12 for Cav3.2KO). (f) Percentage of time spent exploring a familiar object versus a novel object (two-way ANOVA with Bonferroni's multiple comparisons test, *F*_1,26 (interaction)_ ​= ​0.28, *p* ​= ​0.6022, η^2^ ​= ​0.0037) and (g) discrimination index (unpaired *t*-test, *t*_(13)_ ​= ​0.37, *p* ​= ​0.7151, *Cohen's d* ​= ​0.194) in the 24 ​h interval protocol of the NORT after 30 ​min of clonazepam treatment (*n* ​= ​7 for WT; *n* ​= ​8 for Cav3.2KO). (h) Percentage of freezing behavior during the contextual test after 30 ​min of clonazepam treatment (*n* ​= ​10 for WT; *n* ​= ​7 for Cav3.2KO; unpaired *t*-test, *t*_(15)_ ​= ​1.86 *p* ​= ​0.0834, *Cohen's d* ​= ​0.392). ∗*p* ​< ​0.05, ∗∗∗*p* ​< ​0.001 and ∗∗∗∗*p ​<* ​0.0001.

### The GABA level was decreased in the frontal cortex of Cav3.2KO mice

**Fig. 4:**
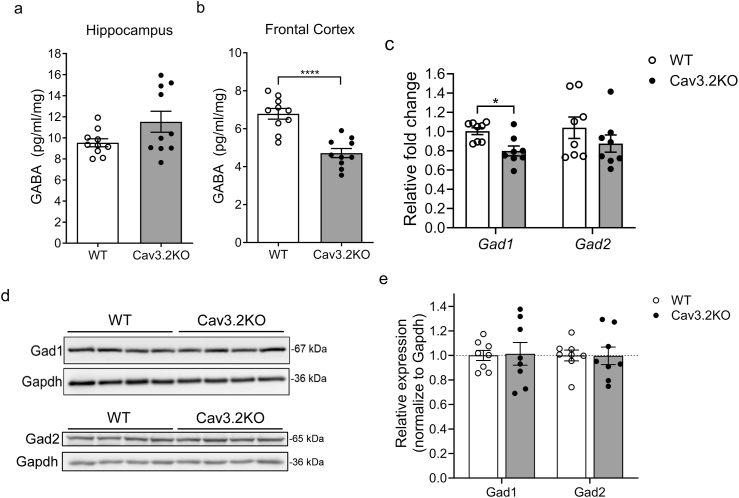
**The expression levels of GABA and GABA-related genes in Cav3.2KO and WT mice** (a) Quantification of the GABA concentration in the hippocampal lysate (unpaired *t*-test, *t*_(18)_ ​= ​1.88, *p* ​= ​0.0770, *Cohen's d* ​= ​0.839) and (b) frontal cortical lysate (unpaired *t*-test, *t*_(18)_ ​= ​5.62, *p* ​< ​0.0001, *Cohen's d* ​= ​2.512) (*n* ​= ​10 for WT; *n* ​= ​10 for Cav3.2KO). (c) Quantitative results of *Gad1* (Mann-Whitney test, *p* ​= ​0.0104) and *Gad2* (unpaired *t*-test, *t*_(14)_ ​= ​1.15, *p* ​= ​0.2687, *Cohen's d* ​= ​0.576) mRNA expression level by qRT-PCR in frontal cortical lysate (*n* ​= ​8 for WT; *n* ​= ​8 for Cav3.2KO). (d) Weston blots of Gad1, Gad2, and Gapdh. (e) Quantitative results of Gad1 (unpaired *t*-test, *t*_(14)_ ​= ​0.13, *p* ​= ​0.8964, *Cohen's d* ​= ​0.064) and Gad2 (unpaired *t*-test, *t*_(14)_ ​= ​0.04, *p* ​= ​0.9672, *Cohen's d* ​= ​0.021) protein expression levels in frontal cortical lysate (*n* ​= ​8 for WT; *n* ​= ​8 for Cav3.2KO). ∗*p* ​< ​0.05 and ∗∗∗∗*p ​<* ​0.0001.

Since enhancement of GABAergic transmission rescue the behavioral deficits observed in Cav3.2KO mice and Cav3.2 channels are known to be expressed in GABAergic interneurons [40,52,53], we hypothesized that GABAergic signaling is impaired in the Cav3.2KO mice. To address this hypothesis, we measured the GABA levels in protein lysates derived from the FC and hippocampus of both Cav3.2KO mice and WT mice using ELISA. The results indicated that GABA levels significantly reduced in the FC of Cav3.2KO mice compared to WT controls, whereas GABA levels in the hippocampus remained comparable between the two groups (Fig. 4a and b). The biosynthesis of GABA is catalyzed by glutamate decarboxylase (GAD), which has two isoforms in the central nervous system: GAD1 and GAD2 [54]. The mRNA expression level of *Gad1* was reduced in the frontal cortex of Cav3.2KO mice (Fig. 4c). However, the corresponding protein levels of Gad1 and Gad2 remained unchanged (Fig. 4d and e).

## Discussion

In this study, we demonstrated that Cav3.2KO mice display ASD-like behaviors, as evidenced by impaired social novelty in the 3CT and increased self-grooming behavior. Besides, Cav3.2KO mice exhibited deficits in novel object recognition, memory performance, and fear memory retrieval. Notably, these behavioral abnormalities were rescued by boosting GABAergic transmission with a low-dose of clonazepam. Furthermore, we found that GABA levels were reduced in the FC of Cav3.2KO mice. Collectively, these findings suggest that impaired GABAergic signaling, resulting from the loss of the Cav3.2 channel, may underlie the observed behavioral deficits. Our results also indicate that CLZ may represent a promising therapeutic method for addressing Cav3.2-related ASD symptoms.

In this study, we demonstrated that adult Cav3.2KO mice exhibit deficits in social novelty in 3CT, consisted with findings from Jiao et al. [35]. Notably, their study used juvenile Cav3.2KO mice (3–4 weeks old), suggesting that ASD-like behavior in Cav3.2KO mice may emerge early in development and persist into adulthood. Recently, Antunes et al. [36] reported that both male and female Cav3.2KO mice display impaired social novelty in both juvenile and adult stages, indicating that this phenotype is a sex-independent. Additionally, we observed that elevated self-grooming in Cav3.2KO mice, in agreement with previous findings [35]. However, another study reported that this phenotype was present only in female Cav3.2KO mice [36]. These discrepancies may stem from differences in experimental conditions, such as the use of offspring from dams intraperitoneally injected with a single dose of saline (10 ​mL/kg) at embryonic day E12.5, or variations in behavioral testing apparatus [36].

In addition, we further demonstrated that Cav3.2KO mice displayed impaired recognition function, memory, and fear memory retrieval, consisted with previous studies [[32], [33], [34]], indicating that the Cav3.2 channel plays a critical role in recognition and memory processes. Interestingly, Cav3.2KO mice showed normal spatial learning and memory in the Morris water-maze [33] and Y-maze tests [32,36], implying that Cav3.2 channels contribute specifically to certain domains of learning and memory. Remarkably, the behavioral impairments observed in Cav3.2KO mice align with phenotypes reported in several well-accepted ASD mice models, including *Scn1a*^*+/−*^ [25], BTBR T+ tf/J [26], and *Arid1b*^*+/−*^ [28] mice, further supporting the validity of Cav3.2KO mice as a relevant model for ASD.

T-type calcium channels are activated and conduct calcium influx in response to subthreshold membrane depolarizations between −65 and −50 ​mV in neurons. Their low threshold for voltage activation, coupled with their tonic inactivation near the resting membrane potential, enables these channels to deactivate and underlie the low-threshold spike and rebound bursting phenomenon in neurons [55,56]. Previous studies demonstrated that dorsal root ganglion neuron from Cav3.2KO mice exhibit a decreased frequency of miniature excitatory postsynaptic currents (mEPSCs) [41] and mature granule cells in the dentate gyrus of these mice displayed impaired burst firing, resulting in reduced synaptic plasticity [57]. Additionally, we found that Cav3.2KO mice exhibit a loss of late-phase long-term potentiation [33] and reduced dendritic spine density [34]. These findings suggest that Cav3.2 channel play a critical role in neuronal excitability.

The Cav3.2 channel is widely expressed throughout the central nervous system, particularly in the hippocampus, amygdala, thalamus, layer V of neocortex, cerebellum and midbrain [39,58,59]. Notably, Cav3.2 channels are expressed in specific populations of GABAergic interneurons, including somatostatin-positive (SST^+^) [52] and parvalbumin-positive (PV^+^) [40,53] cells. Furthermore, selective knockout of the Cav3.2 channel in vesicular GABA transporter (Vgat)-expressing neurons in the arcuate nucleus of the hypothalamus reduces their neuronal firing activity, as evidenced by decreased fire frequency and hyperpolarized resting membrane potential [60]. In addition, T-type calcium channels contribute to GABA release in granule cells of the olfactory bulb [61] and play a role in GABAergic transmission in pyramidal cells of prefrontal cortex (PFC) [62]. Moreover, the Cav3.2 channel is implicated in low-threshold exocytosis via the existence of a syntaxin-1A/Cav3.2 channel complex in central neurons [63]. Taken together, these findings suggest that loss of Cav3.2 channel function may impair presynaptic GABAergic transmission by decreasing the excitability of GABAergic interneurons, especially in the PFC.

The role of hippocampal‒prefrontal interactions in normal brain function is essential for performing cognitive functions, emotional behaviors, working memory, and episodic memory as discussed in review articles [64,65]. In this study, we demonstrated that the GABA levels are reduced in the FC of the Cav3.2KO mice, suggesting an E/I imbalance in this brain region. Notably, PV^+^ interneurons in the PFC and hippocampus play a vital role in regulating social recognition and memory [[66], [67], [68]] and contribute to the pathophysiology of several major neurological disorders, including schizophrenia, bipolar disorder, Alzheimer's disease, and ASD [69]. In addition, the Cav3.2 channels are also present in PV^+^ neuron in the brain [40]. Interestingly, Shen et al. [53] selectively inhibited Cav3.2 channels function in PV^+^ interneurons in the medial PFC (mPFC) by injecting a lentivirus expressing a Cre-dependent Cav3.2 inhibitory peptide into mPFC of PV-Cre mice. The treated mice exhibited multiple behavioral deficits, including impaired spatial learning, reduced social interaction and social recognition deficits, while their anxiety levels remained unaffected. These behavioral phenotypes are strikingly similarity to those observed in our Cav3.2KO mice.

Furthermore, a reduced proportion of c–FOS–positive PV^+^ interneurons in the mPFC was observed in these mice following social interactions, suggesting that blocking Cav3.2 channel function in PV^+^ interneuron decreases the neuronal activity in response to social stimuli. Additionally, specific upregulation of *Cacna1h* transcription in PV interneurons of the mPFC was shown to rescue behavioral deficits in mice with *FMR1 autosome homolog 1* deletion selectively in mPFC PV ​^+^ ​interneurons, which display impaired spatial learning, sociability deficits, and defective sensorimotor gating [53]. Together, these findings indicated that Cav3.2 channel plays a pivotal role in modulating PV^+^ interneuron activity. However, further investigation is required to elucidate how the Cav3.2 channel affects the electrophysiological properties of PV^+^ interneuron in the mPFC.

In this study, we demonstrate that enhancing GABAergic transmission via administration of low-dose CLZ rescues behavioral deficits in Cav3.2KO mice. Similarly, CLZ has been shown to ameliorate behavioral deficits in several ASD mouse models, including genetic [25,27,28], environment-induced [29], and idiopathic [26] models. Together, these findings strongly suggest that enhancing GABAergic signaling may improve behavioral outcomes by compensating a potentially excessive glutamatergic neurotransmission, particularly through the use of benzodiazepines (BZDs) [70]. This supports the idea that BZDs, such as CLZ, represents a promising therapeutic strategy not only for individuals with ASD carrying loss-of-function *CACNA1H* variants, but also for other ASD patient with underlying GABAergic dysregulation. Consistent with this viewpoint, a Phase II clinical trial (NCT01966679, registration date: 2013-10-21, https://clinicaltrials.gov/study/NCT01966679) and a Phase II open-label pilot study (NCT01881737, registration date: 2011-11-28, https://clinicaltrials.gov/study/NCT01881737) were conducted to evaluate the efficacy of enhancing GABA_A_Rs-mediated transmission in improving behavioral symptoms in adults with ASD. However, the potential adverse effects associated with long-term BZDs use must be carefully considered [71,72]. Therefore, further well-designed clinical trials are needed to clarify the therapeutic benefits and optimize treatment strategies targeting GABAergic pathways in ASD.

In addition to ASD, recent studies have reported that loss-of-function *CACNA1H* variants are implicated in several neurological disorders, including body-wide chronic pain [73], severe infantile-onset amyotrophy [74], and amyotrophic lateral sclerosis [75,76]. These findings suggest that loss of Cav3.2 channel function contributes to not only psychiatric conditions but also plays an essential role in somatosensory processing [77,78] and muscular development [79,80].

GABA also plays a crucial role in neuronal maturation and activity-dependent circuit integration by triggering excitatory inputs that drive large-scale spontaneous electrical activity in both the central and peripheral nervous systems during early brain development [81]. Thus, dysregulation of GABAergic signaling during neurodevelopment has been linked to several neurological diseases, including ASD [82], schizophrenia [83], attention-deficit/hyperactivity disorder [84], and neonatal seizures [85,86]. Moreover, GABA-induced membrane depolarization can activate VGCCs, which regulate key aspects of neuronal development, including the proliferation and differentiation of neuronal progenitor cells, neuronal migration and synaptogenesis [87]. Notably, Rebellato et al. [88] demonstrated that *Cacna1h* mRNA levels was dramatically upregulated in mouse embryonic stem cells during neuronal differentiation. Additionally, Cav3.2KO mice exhibited reduced spontaneous Ca^2+^ activity in the cortical region, along with a diminished cortical plate and ventricular zone. These findings suggest that excitatory GABAergic signaling and Cav3.2 channels converge on shared Ca^2+^-dependent developmental phenomena.

Interestingly, Antunes et al. [36] demonstrated that prenatal exposure to valproic acid (VPA), an antiepileptic drug, rescues impaired sociability in Cav3.2KO mice. Although the mechanism of action of VPA is not fully comprehended, one of suggested mechanisms of action is enhancing GABAergic signaling through inhibition of GABA transaminase and promotion of GABA synthesis [89]. Therefore, boosting GABAergic signaling with anticonvulsant drug may represent a potential therapeutic strategy for various neurological disorders associated with GABAergic dysfunction [90,91].

Surprisingly, our results demonstrated that the Cav3.2KO mice displayed normal behaviors in both the EPM test and OFT compared to WT mice. In contrast, a previous study showed that the Cav3.2KO mice spent less time in the open arms of the EPM and the center of the OFT, suggesting the Cav3.2KO mice exhibit anxiety-like behavior [32]. The discrepant findings may be attributed to differences in experimental parameters, such as the height of the EPM apparatus (100 ​cm above the floor in Ref. [32] vs. 60 ​cm in our study) and the definition of the center zone in the OFT (57 ​% of total area in Ref. [32] vs. 11 ​% in our study). On the other hand, marble burying test is employed as a model to study anxiety- and compulsive-like behaviors [92]. In our study, WT mice displayed high levels of marble-burying behavior, consistent with previous studies [93,94]. In contrast, Cav3.2KO mice presented a significant reduction in the number of marbles buried (Fig. 2f). This observation aligns with findings from another ASD mouse model, the *Shank2*^−/−^ mice, which also display decreased marble-burying behavior [95]. Notably, administration of midazolam, a type of benzodiazepine, did not alter marble-burying behavior in *Shank2*^−/−^ mice, suggesting that enhancing GABAergic transmission has limited effects on rescuing this specific behavioral phenotype in both *Shank2*^−/−^ mice and Cav3.2KO mice (Supplementary Fig. 4g).

Since ASD can be driven by multiple factors, including environmental pollutants and mutations in specific genes, it is very difficult to use a type of mouse model to study all aspects of ASD pathology. Thus, our findings are most applicable to individuals with ASD carrying the *CACNA1H* variants and may not generalize to all forms of ASD. Additionally, our study used only one strain of mouse, while species-specific differences may occur. Experiments were primarily conducted using adult male mice to minimize variability associated with estrous cycles. Although sex and timepoint were not variables examined in this work, we acknowledge them as important biological factors. Future studies will be necessary to determine whether the observed effects are sex- and time-dependent. Moreover, Gad1 protein levels remained unchanged despite reduced *Gad1* mRNA expression, suggesting that multiple factors may influence Gad1 regulation, including epigenetic mechanisms, glutamate and N-methyl-D-aspartic acid receptor (NMDAR) signaling, BDNF–TrkB–RAS–ERK–CREB signaling, and post-transcriptional modifications of GAD [96]. Further *in vitro* and *in vivo* studies are warranted to elucidate these mechanisms.

In conclusion, we found that Cav3.2KO mice exhibit reduced GABA levels in the FC, along with impairment in social novelty, recognition and memory functions, as well as increased self-grooming. These findings suggest that Cav3.2 channel loss-of-function contributes to E/I imbalance and underlies core behavioral phenotypes associated with ASD. To our knowledge, this is the first study to demonstrate that enhancing GABAergic transmission with low-dose CLZ rescues the behavioral impairments in Cav3.2KO mice. Taken together, our results support targeting GABAergic signaling as a potential therapeutic strategy for individuals with ASD carrying loss-of-function *CACNA1H* variants and highlight the prospective utility of BZDs in this context.

## Consent of publication

Not applicable.

## Ethics approval

All protocols used in this study have been reviewed and approved by the Institutional Animal Care and Use Committee of Tzu Chi University (TCU, #108029). All the experimental procedures followed the Taiwan Ministry of Science and Technology guidelines for the ethical treatment of animals.

## Availability of data and materials

The datasets used and/or analyzed during the current study are available from the corresponding author upon reasonable request.

## Authors contributions

S.F.L., Y.M., R.Y.L., I.Y.L. and C.C.C. contributed to the hypothesis development, and research design. S.F.L. and Y.M. performed the experiments, and data analysis. I.Y.L., C.C.C., S.C and S.F.L. wrote the manuscript. H.T.H. was responsible for the breeding and genotyping of the mice. All the authors read and approved this manuscript.

## Declaration of generative AI and AI-assisted technologies in the writing process

During the preparation of this work the authors used ChaTGPT and Gemini in order to improve the readability and language of the article. After using these tools, the authors reviewed and edited the content as needed and take full responsibility for the content of the publication.

## Funding

This study was supported by grants from the Buddhist Tzu Chi Medical Foundation (Grant #: TCMF-SP 112-02), and the National Science and Technology Council (NSTC), Taiwan (Grant #: NSTC 112-2410-H-320-004 and NSTC 113-2410-H-320-004-MY2) to I.Y.L. and Academia Sinica grants (Grant #: AS-IR-(111–113)-05-A), Taiwan Ministry of Science and Technology grants (Grant #: MOST 111-2320-B-001-007-MY3) to C.C.C.

## Declaration of Competing Interest

The authors declare that they have no competing interests.

## Figures and Tables

**figs1:**
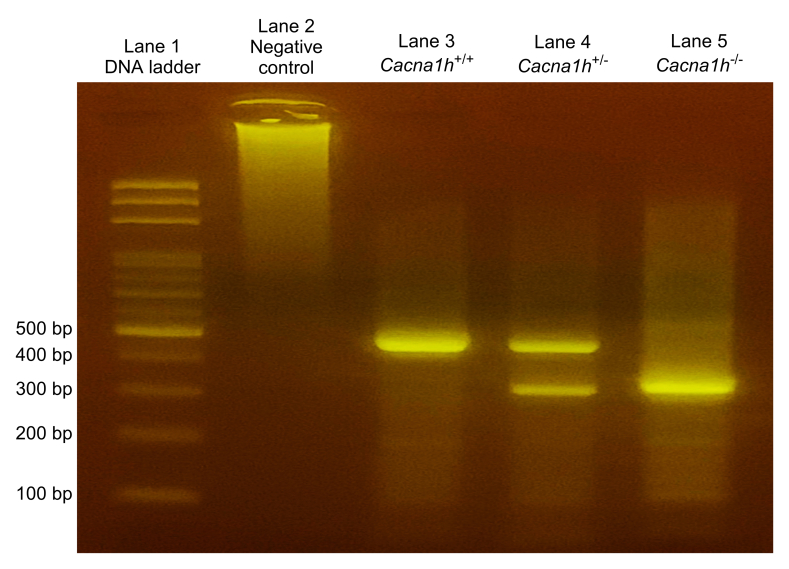


**figs2:**
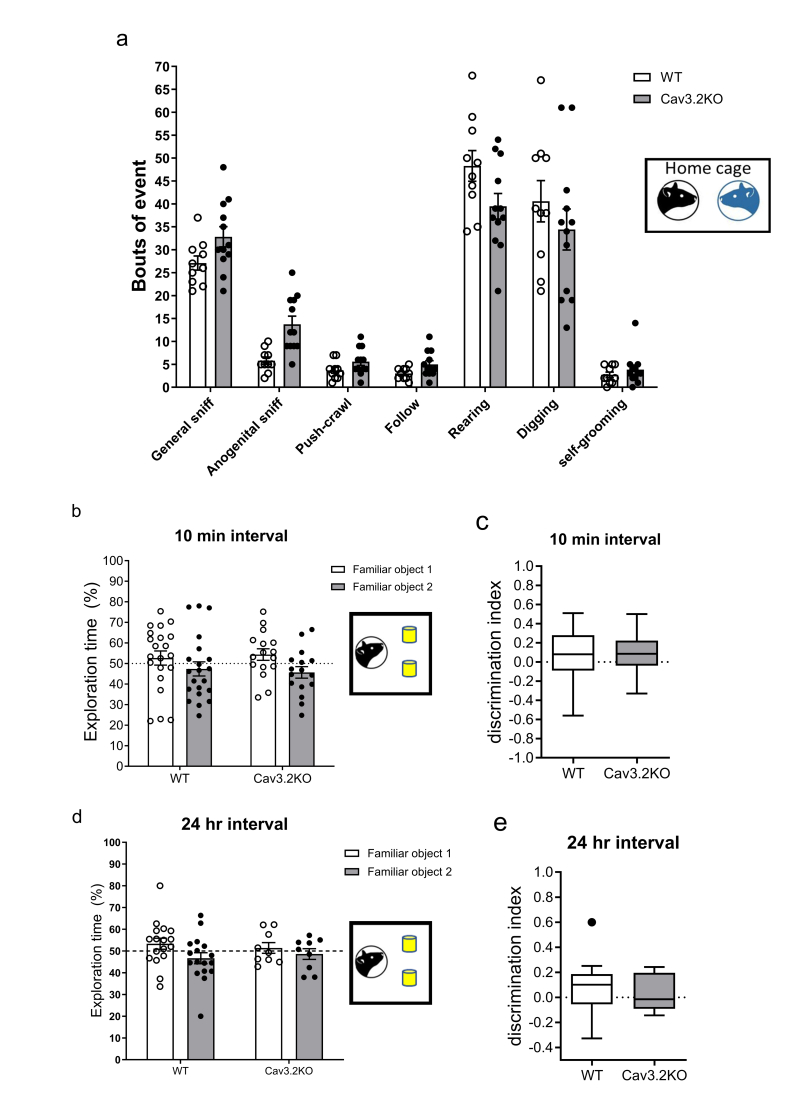


**figs3:**
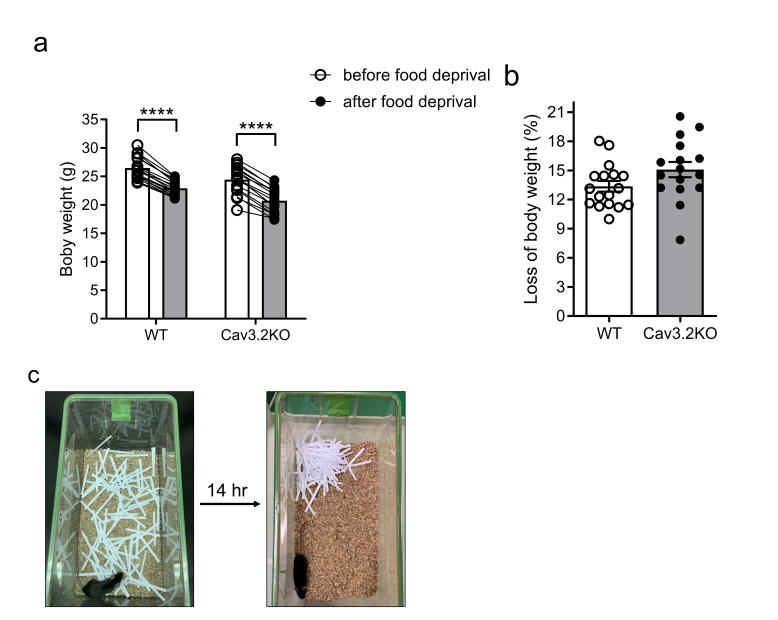


**figs4:**