# Supplementary material for: Activation of GABA transmission by clonazepam reverses the autistic-like phenotypes of the Cav3.2 knockout mice
Source: Neurotherapeutics. 2025 Oct 8;22(6):e00761. doi: 10.1016/j.neurot.2025.e00761 (PMC12664563; doi:10.1016/j.neurot.2025.e00761)
Supplement: Multimedia component 1 [file mmc1.docx]

**Supplementary materials**

**Supplementary Fig. 1** Genotypes of WT littermate, heterozygous littermate and Cav3.2KO mice were identified by PCR. A DNA ladder is shown in the first lane. The negative control (ddH₂O) is placed in the second lane. The WT littermate displays a single band at 480 bp in the third lane. The heterozygous littermate shows two bands at 480 bp and 330 bp in the fourth lane. The Cav3.2KO mouse exhibits a single band at 330 bp in the fifth lane.

**Supplementary Fig. 2** (a) The bouts of non-social behaviors (digging, rearing and self-grooming) and social behaviors (general sniffing, anogenital sniffing, following and push-crawl) in the reciprocal social interaction test (*n* =10 for WT; *n* =12 for Cav3.2KO; two-way ANOVA with Bonferroni's multiple comparisons test, *F* _6,140 (interaction)_ = 3.39, *p* = 0.0038, η² = 0.024). (b) Percentage of time spent exploring a familiar object 1 versus a familiar object 2 (two-way ANOVA with Bonferroni's multiple comparisons test, *F* _1,70 (interaction)_ = 0.27, *p* = 0.6037, η² = 0.036) and (c) discrimination index (unpaired t-test, *t*_(35)_ = 0.34, *p* = 0.7341, *Cohen’s d* = 0.116) in the 10 min interval protocol of the NORT (*n* =21 for WT; *n* =16 for Cav3.2KO). (d) Percentage of time spent exploring a familiar object 1 versus a familiar object 2 (two-way ANOVA with Bonferroni's multiple comparisons test, *F* _1,48 (interaction)_ = 0.49, *p* = 0.4868, η² = 0.010) and (e) discrimination index (unpaired t-test, *t*_(24)_ = 0.50, *p* = 0.6248, *Cohen’s d* = 0.215) in the 24 h interval protocol of the NORT (*n* =17 for WT; *n* =9 for Cav3.2KO).

**Supplementary Fig. 3** (a) Average body weights of the mice before and after 20 h food deprivation (*n* =17 for WT; *n* =16 for Cav3.2KO; two-way repeated measures ANOVA with Bonferroni's multiple comparisons test; *F* _1,31 (interaction)_ = 0.20, *p* = 0.6573, η² = 0.0001). (b) Percentage of weight loss in WT and the *Ca_v_3.2KO* mice after 20 h food deprivation (*n* =17 for WT; *n* =16 for Cav3.2KO; unpaired t-test, *t*_(31)_ = 1.82, *p* = 0.0792, *Cohen’s d* = 0.630). (c) Representative images from the nest building test. *****p* < 0.0001.

**Supplementary Fig. 4** (a) Total distance moved of WT mice in OFT after 30 min of CLZ injection compared to saline control (*n* =9 for WT+saline; *n* =9 for WT+CLZ; unpaired t-test, *t*_(16)_ = 1.66, *p* = 0.1158, *Cohen’s d* = 0.784). (b) Percentage of time spent in the closed arms of the EPM in WT mice after 30 min of CLZ injection compared to saline control (*n* =6 for WT+saline; *n* =9 for WT+CLZ; unpaired t-test, *t*_(13)_ = 0.63, *p* = 0.5370, *Cohen’s d* = 0.320). (c) Percentage of time spent exploring a familiar object 1 versus a familiar object 2 (two-way ANOVA with Bonferroni's multiple comparisons test, *F* _1,42 (interaction)_ = 6.82, *p* = 0.0124, η² = 0.139) and (d) discrimination index (unpaired t-test, *t*_(21)_ = 1.84, *p* = 0.0805, *Cohen’s d* = 0.758) in the 10 min interval protocol of the NORT after 30 min of clonazepam treatment. (*n* =11 for WT, *n* =12 for Cav3.2KO). (e) Percentage of time spent exploring a familiar object 1 versus a familiar object 2 (two-way ANOVA with Bonferroni's multiple comparisons test, *F* _1,26 (interaction)_ =3.077×10^−5^, *p* = 0.9956, η² = 1.12×10^−6^) and (f) discrimination index (unpaired t-test, *t*_(13)_ = 0.004, *p* = 0.9969, *Cohen’s d* = 0.002) in the 24 h interval protocol of the NORT after 30 min of clonazepam treatment (*n* =7 for WT, *n* =8 for Cav3.2KO). (g) Number of marbles buried after 30 min of clonazepam treatment (*n* =6 for WT, *n* =3 for Cav3.2KO; Mann-Whitney test, *p* = 0.0357). **p* < 0.05.
